# Diversity of Yeasts and Molds by Culture-Dependent and Culture-Independent Methods for Mycobiome Surveillance of Traditionally Prepared Dried Starters for the Production of Indian Alcoholic Beverages

**DOI:** 10.3389/fmicb.2018.02237

**Published:** 2018-09-26

**Authors:** Shankar Prasad Sha, Mangesh Vasant Suryavanshi, Kunal Jani, Avinash Sharma, Yogesh Shouche, Jyoti Prakash Tamang

**Affiliations:** ^1^DAICENTRE (DBT-AIST International Centre for Translational and Environmental Research) and Bioinformatics Centre, Department of Microbiology, School of Life Sciences, Sikkim University, Gangtok, India; ^2^National Centre for Microbial Resource, National Centre for Cell Science, Pune, India

**Keywords:** mycobiome, dried starters, PCR-DGGE analysis, yeasts, filamentous molds

## Abstract

*Marcha, thiat, dawdim, hamei, humao, khekhrii, chowan*, and *phut* are traditionally prepared dried starters used for production of various ethnic alcoholic beverages in North East states of India. The surveillance of mycobiome associated with these starters have been revealed by culture-dependent methods using phenotypic and molecular tools. We identified *Wickerhamomyces anomalus, Pichia anomala, Saccharomycopsis fibuligera, Pichia terricola, Pichia kudriavzevii*, and *Candida glabrata* by ITS-PCR. The diversity of yeasts and molds in all 40 samples was also investigated by culture-independent method using PCR-DGGE analysis. The average distributions of yeasts showed *Saccharomyces cerevisiae* (16.5%), *Saccharomycopsis fibuligera* (15.3%), *Wickerhamomyces anomalus* (11.3%), *S. malanga* (11.7%), *Kluyveromyces marxianus* (5.3%), *Meyerozyma* sp. (2.7%), *Candida glabrata* (2.7%), and many strains below 2%. About 12 strains of molds were also identified based on PCR-DGGE analysis which included *Aspergillus penicillioides* (5.0%), *Rhizopus oryzae* (3.3%), and sub-phylum: *Mucoromycotina* (2.1%). Different techniques used in this paper revealed the diversity and differences of mycobiome species in starter cultures of India which may be referred as baseline data for further research.

## Introduction

Essence of alcoholic fermentation depends on different types of starters that copulate the uniqueness to organoleptic segmentations for ethnic values (Hesseltine, 1983; Steinkraus, 1996; Tamang et al., 2016b). Yeasts have several economic significances and have been used for centuries in the production of fermented foods and alcoholic beverages (Fleet, 2003; Tamang and Fleet, 2009; Jolly et al., 2017). In Asia, preparation of amylolytic (related to conversion of starch to sugar) and alcoholic (production of alcohol) starter is an innovative back sloping technique of cultivation of native microbiota in the form of dry, flattened, or round balls made up of rice/wheat for production of different traditional alcoholic beverages (Tamang, 2010), locally known as *marcha* in India, Nepal and Bhutan, *benh men* in Vietnam, *bubod* in the Philippines, *chiu/chu/daque* in China and Taiwan, *loogpang* in Thailand, *ragi* in Indonesia, and *nuruk* in Korea (Tamang, 2016). Traditional methods of preparation of Asian amylolytic dry starters are similar with slight variation in terms of wrapping materials, incubation period, size, and shapes of particular starters. Ethnic people practicing the age-old traditional preservation or sub-culturing amylolytic and alcohol-producing as well as flavor-enhancing fungi and bacteria have attracted many researchers to study the microbial diversity in such starters. In recent years, few researchers have reported the fungal and bacterial species using both culture-dependent and-independent techniques in some common starter cultures of Asia such as *marcha* of India (Tsuyoshi et al., 2005; Sha et al., 2017), *daqua* of China (Wang et al., 2008; Zheng et al., 2012; Lv et al., 2013; Chen et al., 2014; Xu et al., 2017), *benh men* of Vietnam (Dung et al., 2007; Thanh et al., 2008); *nuruk* of Korea (Jung et al., 2012), and *dombea* of Cambodia (Ly et al., 2018).

**FIGURE 1:**
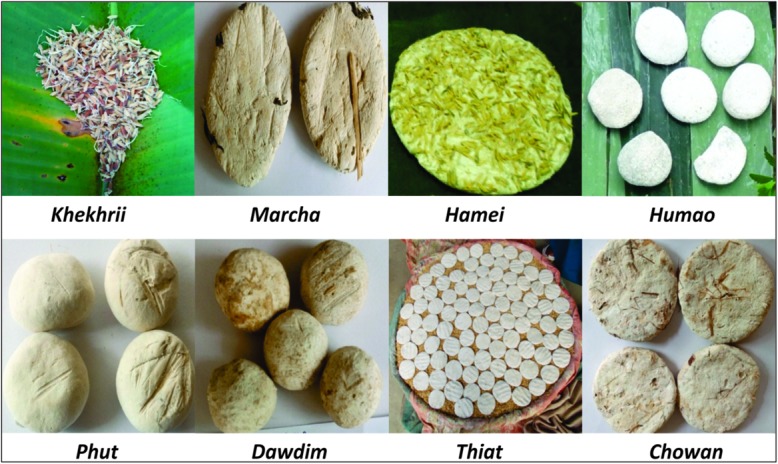
Traditionally prepared dried starters collected from different parts of North East India.

North East regions of India^1^ have several varieties of traditionally prepared and sun-dried starters prepared by different linguistic ethnic groups of people that include *marcha* of Sikkim, *humao* of Assam, *hamei* of Manipur, *chowan* of Tripura, *thiat* of Meghalaya, *khekhrii* of Nagaland, *dowdim* of Mizoram, and *phut* of Arunachal Pradesh (**Figure 1**). These starter cultures except *khekhrii* of Nagaland are traditionally prepared from soaked rice with some wild herbs, and then mixed with previously prepared starter powder (1–2%) as an inoculum (back-sloping). The mixtures are ground in a wooden mortal with addition of water to make a thick dough which are kneaded into round to flattened balls/cakes of different size and shape. Dough cakes are covered with fern fronds/paddy straws/jute sags, fermented at room temperature for 1–3 days; and fresh balls/cakes are sun dried for few days (Anupma et al., 2018). *Khekhrii* of Nagaland is prepared by naturally fermenting sprouted-rice grains and then sun-dried to use as dry starter culture to prepare *zutho*, local alcoholic beverage. Some species of yeasts *Saccharomycopsis fibuligera, S. capsularis, Pichia anomala, P. burtonii, P. guilliermondii, P. fabianii, Trichosporon* sp., *Candida tropicalis, C. parapsilosis, C. montana, C. glabrata, Torulaspora delbrueckii, Saccharomyces cerevisiae, S. bayanus*, and *Wickerhamomyces anomalus* were previously reported from some samples of *marcha* and *hamei* of India (Hesseltine and Kurtzman, 1990; Tamang and Sarkar, 1995; Tsuyoshi et al., 2005; Jeyaram et al., 2008, 2011, Sha et al., 2016, 2017).

One of the common methods for culture-dependent identification is by the analysis of the Internal Transcribed Spacer (ITS)1-5.8S-ITS2 region, which is widely applied in explorations of diversity of fungi associated with many traditional fermented foods (Caggia et al., 2001; Las Heras-Vazquez et al., 2003). ITS analysis may provide the fast and easy means for accurate identification at species level (Esteve-Zarzoso et al., 1999), due to greater sequence variation, the ITSl/ITS2 domains are more suited for species and strain identification than the 18s region (small subunit), the 5.8s region, and the 28s region (large subunit) (Iwen et al., 2002; Korabecna, 2007; Susan Slechta et al., 2012). However, the culture-dependent methods may not detect the whole microbial community in foods (Ercolini, 2004). The culture-independent methods such as PCR denaturing gradient gel electrophoresis (DGGE) analysis, are highly useful to detect the whole microbial communities in food samples (Chen et al., 2014; Puerari et al., 2015; Tamang et al., 2016a). PCR-DGGE analysis method has been designed to profile microbial communities directly from substrates including fermented foods, and is based on sequence-specific distinctions of 16SrRNA and 26SrRNAmplicons (Cocolin et al., 2000; Ercolini, 2004; Ercolini et al., 2004; Alegría et al., 2011).

No studies have been conducted on traditionally prepared starters of India except *marcha* (Tamang and Sarkar, 1995; Tsuyoshi et al., 2005; Sha et al., 2016, 2017), and *hamei* (Tamang et al., 2007; Jeyaram et al., 2008, 2011). Based on our preliminary analysis of microbial load in traditionally prepared starters of North East India, fungi mostly yeasts and filamentous molds (>10^6^ cfu/g) predominate over bacteria. Hence, we aimed to study the mycobiome diversity in dried starters of India by culture-dependent and -independent methods to underline the continuous interest in the characterization of microbial consortia associate to poorly studied food fermentations to isolate new potential pro-technological and functional strains, to improve the conservation of microbial diversity, to characterize and limit spoilage microbes, microbial producers of toxic compounds, and pathogens (Capozzi and Spano, 2011; Russo et al., 2016; Tamang et al., 2016a,b; Gonelimali et al., 2018).

## Materials and Methods

### Sample Collection

Forty different samples of traditionally prepared starter (five samples of each starter) *marcha* of Sikkim, *thiat* of Meghalaya, *hamei* of Manipur, *phut* of Arunachal Pradesh, *chowan* of Tripura, *dawdim* of Mizoram, *humao* of Assam, and *khekhrii* of Nagaland were collected immediately after the preparation (fermentation and sun-dried drying) from local people of eight states of North East India, and were transferred to gamma irradiated sterile bottles, sealed, and stored in desiccator at room temperature for the further analysis.

### Isolation of Microorganisms

Ten grams of sample was homogenized with 90 ml of 0.85% (w/v) sterile physiological saline in a stomacher lab-blender 400 (Seward, United Kingdom) for 1 min and serially diluted in the same diluents. Yeasts were isolated on yeast-malt extract agar (M424, HiMedia, India) and molds were isolated on potato dextrose agar (M096, HiMedia, India) supplemented with 10 IU ml^-1^ benzyl penicillin and 12 mg ml^-1^ streptomycin sulfate, and were incubated aerobically at 28°C for 3 days. Purity of the isolates was checked by streaking again on fresh agar plates of the same isolation medium, followed by microscopic examination. Isolation of yeast strains were typically based on morphotypes and criterion included size, color, shape, and appearance of fully grown culture on growth media. Colonies were counted as colony forming units (cfu)/g sample. Identified strains of yeasts were preserved in 20% glycerol at -20°C (Thapa and Tamang, 2004).

### Culture-Dependent Approach for Diversity Analysis

#### Phenotypic and Biochemical Characterization

A total of 386 yeasts strains were isolated from 40 samples of eight different starters of North East India. Characterizations of yeasts were phenotypically tested on the basis of colony and cell morphology, sugar fermentation, and sugar assimilation tests. Cell morphology of actively growing yeast isolates was determined using a phase-contrast microscope (CH3-BH-PC; Olympus, Tokyo, Japan). Yeast cultures have been characterized on the basis of mycelium type, ascospore type, nitrate reduction, growth at 37 and 45°C, sugar fermentation, and sugar assimilation following the methods of Kurtzman et al. (2011).

#### Biolog System

Commercial Biolog Identification System (MicroLog TM System Release 4.2 User Guide 2001, Biolog, Inc.) based on the utilization of 95 substrates in 96-welled plate, were used for biochemical characterization of yeast isolates. Aliquots of the cultures were transferred to biolog plate wells and incubated at 37°C for 24–48 h, where positive results were recorded according to color changes. The results obtained were automatically read and analyzed using BiologMicrolog Reader and compared with the database of the Biolog Microlog database software (Biolog Inc.), which provided the most probable genera and species of the tested cultures.

#### Molecular Identification of Yeast Isolates

Identification of yeast isolates were done by ITS region sequencing wherein DNA extraction, PCR for ITS region, sequencing, and phylogenic affiliations were performed subsequently. Briefly, yeast DNA was extracted using ProMega DNA kit (ProMega). One gram of yeast cell pellet was suspended in lysis solution and incubated at 65°C for 15 min. Subsequently, the RNA was eliminated from the cellular lysate by administering the RNase solution following incubation at 35°C for 15 min. The residual proteins were removed by adding protein precipitation solution and centrifugation at maximum speed. Finally, the DNA was precipitated by adding isopropanol, which was purified with two washes of 70% ethanol. The quality of DNA was checked on 0.8% agarose gel and concentration was measured using Nano-Drop ND-1000 spectrophotometer (Nano Drop Technologies, Wilmington, DE, United States) as described by Banskar et al. (2016). The DNA was stored at -20°C until further processing. For amplification of the ITS region, the forward primer ITS1 (5^′^-TCCGTAGGTGAACCTGCGG-3^′^) and reverse primer ITS4 (5^′^-TCCTCCGCTTATTGATATGC-3^′^) (White et al., 1990) were used and PCR mixture and the thermal cycling protocol conditions were applied as described by (Esteve-Zarzoso et al., 1999). Products were analyzed on 1.5% agarose gel containing 0.7 mg/ml of ethidium bromide and visualized under UV light (UV source Gel-Doc 1000, Bio-Rad). Approximate size of amplicons was determined using standard molecular weight markers (Himedia-100-bp DNA Ladder) (Lv et al., 2013). All PCR-amplified products were purified and sequenced using ABI-DNA-Sequencer (ABI Genetic Analyser 3500, HITACHI, Japan). The sequences were compared with the GenBank database using the BLAST program (Altschul et al., 1990; Zhao and Chu, 2014). Sequences were visualized and edited using Chromas Version 1.45^2^ (Pryce et al., 2003).

### Culture-Independent Approach for Diversity Analysis

#### DNA Extraction, PCR Amplification From Starter Cultures

About 10 g of starters was homogenized in 90 ml of 0.85% w/v sterile physiological saline, and subsequently filtered through four layers of sterile cheese-cloth. The resulting filtered solutions were centrifuged at 14,000 *g* for 10 min at 4°C (Lv et al., 2013). Then, the pellets were subjected to DNA extraction using the ProMega DNA extraction kit (ProMega, United States) according to the manufacturer’s instructions. Quality of resultant DNA was checked on 0.8% agarose gel and concentration was measured using Nano-Drop ND-1000 spectrophotometer (Nano Drop Technologies, Wilmington, DE, United States) as previously described (Banskar et al., 2016). The 250 nucleotides of the 5^′^-end D1/D2 region of the 26SrRNA gene was amplified by PCR using the primer NL1 (5^′^-CGC CCG CGC GCG GGC GGG GCG GGG GCC ATA TCA ATA AGC GGA AAA G-3^′^) (the GC clamp sequence used is underlined) and a reverse primer LS2 (5^′^-ATT CCC AAA CAA CTC GAC TC-3^′^) (Cocolin et al., 2000; El Sheikha et al., 2009). PCR was performed in a final volume of 50 μl containing 10 mM Tris–HCl, 50 mM KCl, 1.5 mM MgCl_2_, 0.2 mM each dATP, dCTP, dGTP, and dTTP, 0.2 mM of the primers, and 1.25 IU *Taq*-DNA polymerase (Promega, United States) and 2 μl of the extracted DNA (approximately 50 ng) using Thermal Cyclers (Applied Biosystems, United States). The reactions were run for 30 cycles at 95°C for 60 s for denaturation, at 52°C for 45 s for annealing, and at 72°C for 60 s for extension and finally for 7 min at 72°C (Cocolin et al., 2002). The PCR products were analyzed on 2.0% agarose gel containing 0.5 μg/ml ethidium bromide and were visualized in UV source GelDoc (Bio-Rad) (Cocolin et al., 2000). The concentration was again measured using Nano-DropND-1000 spectrophotometer.

#### PCR-DGGE Fingerprinting and Sequencing of DGGE Eluted Bands

The PCR products were analyzed by DGGE using DCode^TM^ Universal Mutation Detection System (DGGEK-1001, CBS Scientific, San Diego, CA, United States) following the procedure of El Sheikha et al. (2009). Samples containing approximately equal amounts of PCR products were loaded into 8% w/v polyacrylamide gels (acrylamide:*N,N*^′^-methylene bisacrylamide, 37.5:1; Promega) in 1 × TAE buffer (40 mM Tris–HCl, pH 7.4, 20 mM sodium acetate, and 1.0 mM Na_2_-EDTA). All electrophoresis experiments were performed at 60°C using a denaturing gradient in the range of 30–50% (100% corresponded to 7 M urea and 40% v/v formamide; Promega) (Cocolin et al., 2002). The gels were electrophoresed at 20 V for 10 min and then at 80 V for 12 h (El Sheikha et al., 2009). The gels were stained with SYBR Gold for 30 min (reconstituted according to the manufacturer’s directions; Molecular Probes, Invitrogen, United States) and photographed in UV source GelDoc (Bio-Rad, United States) as described by Grizard et al. (2014). The DGGE bands were excised using sterile micro pipette tips. DNA of each band was eluted in 50 μl sterile water overnight at 4°C and 2 μl of the eluted DNA was reamplified as following the method of Cocolin et al. (2000). The PCR products which yielded only one band in DGGE electrophoresis were amplified with the primers without GC-clamp, purified and finally sequenced with the help of ABI-DNA-Sequencer (ABI Genetic Analyser 3500, HITACHI, Japan). The sequences were compared with the GenBank database using the BLAST program (Altschul et al., 1990; Zhao and Chu, 2014). The DNA sequences obtained from sequencing of total 202 bands was submitted to GeneBank.

### Bioinformatics and Statistical Analysis

Quality of raw ITS region from yeast isolates and PCR-DGGE band sequencing data was checked with the help of Sequence Scanner software (Applied Bio systems, United States) and the data alignment and analysis were done with the help of SEQMANN software (DNASTAR, United States). After the data alignment, BLAST program was used for comparing DNA databases for sequence similarities available on the server^3^ (Altschul et al., 1990; Zhao and Chu, 2014). Construction of a phylogenetic tree by the neighbor-joining method (Saitou and Nei, 1987) was performed using the CLUSTAL W program (Thompson et al., 1994). Shannon index of general diversity (H) and the richness of the microbial community as microbial diversity indices were determined by following the method of Oguntoyinbo et al. (2011). Other graphical emphasis was done on *igraph* package in R Software (Csardi and Nepusz, 2006).

### Nucleotide Accessions

The sequences obtained from ITS region sequencing of isolated 46 yeast strains have been deposited in the GenBank under accessions: KY587119–KY626335 and 26S rRNA gene of 202 bands excised from PCR-DGGE under accessions: KY594045–KY594246.

## Results

### Culture-Dependent Approach

The average populations of yeast in all eight starters was 7.2 × 10^6^ cfu/g (**Table 1**). Ascertaining the cultured diversity, a total of 386 yeasts strains were isolated from 40 samples and characterized by phenotypic assessment on the basis of colony morphology, cell morphology, sugar fermentation, and sugar assimilation tests (**Table 2**). Tentatively the following yeast genera were phenotypically identified using the taxonomical keys of Kurtzman et al. (2011) as *Saccharomyces, Pichia, Candida, Issatchenkia, Kluyveromyces, Schizosaccharomyces, Saccharomycopsis*, and *Torulopsis* (**Table 2**). Their metabolic capacities were also assessed by using the Biolog system. By comparing with the yeast database (MicroLog TM System Release 4.2 User Guide 2001, Biolog), the result revealed that maximum identified yeast species were associated with starter having ≥0.75% probability and ≥0.7 similarities index value (**Table 3**). The yeasts strain *Pichia terricola* showed highest ≥0.974% probability with ≥0.77 similarities index value. It was observed that the results from Biolog were revealing more diversity of yeasts than phenotypic characterization and it presented in **Supplementary Data Sheet S1**.

**Table 1:** Average populations of yeasts in starters of North East India.

**Samples**	*Marcha*	*Humao*	*Hamei*	*Thiat*	*Phut*	*Khekhrii*	*Chowan*	*Dawdim*
**States**	Sikkim (*n* = 10)	Assam (*n* = 5)	Manipur (*n* = 5)	Meghalaya (*n* = 5)	Arunachal Pradesh (*n* = 5)	Nagaland (*n* = 5)	Tripura (*n* = 5)	Mizoram (*n* = 5)
**Log cfu/g**	6.865 (±0.06)	6.834 (±0.14)	6.852 (±0.03)	6.839 (±0.08)	6.836 (±0.05)	6.851 (±0.04)	6.852 (±0.03)	6.851 (±0.04)

*n, number of samples analyzed; cfu, colony forming unit; standard deviation are given in parenthesis*.

**Table 2:** Grouping of total isolates of yeasts from starters of North East India on the basis of fermentation, and assimilation of sugars and other phenotypic tests.

Parameters	Tentative identity							
	*Saccharomyces*	*Pichia*	*Candida*	*Issatchenkia*	*Kluyveromyces*	*Schizosaccharomyces*	*Saccharomycopsis*	*Torulopsis*
Total isolates	43	60	56	51	41	52	23	60
**Sugar fermented**		
Lactose	+ (3), - (40)	-	+ (6), - (50)	-	-	-	-	-
Raffinose	+ (37), - (6)	+ (56), - (4)	+ (57), - (3)	+	+	+	+ (18), - (5)	+ (56), - (4)
Xylose	+ (39), - (4)	+ (55), - (5)	+ (54), - (6)	+ (45), - (5)	+	+ (50), - (2)	+ (20), - (3)	+ (55), - (5)
**Sugar assimilated**			
Trehalose	+ (40), - (3)	+ (50), - (10)	+ (55), - (5)	+ (46), - (5)	+	+ (50), - (2)	+ (20), - (3)	+ (50), - (10)
Lactose	+ (3), - (40)	+	+ (4), - (52)	-	+	+ (4), - (48)	+ (3), - (20)	-
Raffinose	+ (39), - (4)	+ (51), - (5)	+ (50), - (6)	+ (47), - (4)	+ (38), - (3)	+ (47), - (5)	+ (20), - (3)	+ (56), - (4)
Melibiose	+ (40), - (3)	+ (55), - (5)	+ (54), - (6)	+	+	+	+	+
True/pseudo-mycelia	Pseudo-mycelia	Pseudo-mycelia	True mycelia	Pseudo-mycelia	Pseudo-mycelia	Pseudo-mycelia	Pseudo-mycelia	Pseudo-mycelia
Ascospore	Hat-shaped	Hat-shaped	Oval	Spheroidal	Ellipsoidal	Globose	Hat-shaped	Spheroidal
Representative strains	GM:Y12, AS:Y12, HM:Y15, ST:Y46, AP:Y45, M:Y1, CH:Y22	GM:Y34, AS:Y3, HM:Y3, ST:Y3, AP:Y4, KY:Y3, M:Y49, CHY:34	GM:Y37, AS:Y7, HM:Y7 ST:Y41, AP:Y22, KY:45, MY:47, CHY:37	GM:Y4, AS:Y4, HM:Y50, ST:Y24, AP:Y3,KY:Y4, M:Y3, CHY:36,	GM:Y29, AS:Y6, HM:Y26, ST:Y36, AP:Y6, KY:33, M:Y6, CM:Y10,	AS:Y45, HM:Y9, ST:Y49, AP:Y15, KY:Y5, M:Y9, CH:Y15	GM:Y22, AS:Y2, HMY12, ST:Y12, AP:Y2, KY:Y42, M:Y2, CH:Y22	GM:Y1, AS:Y1, HM:Y28, ST:Y30, AP:Y38, KY:Y10, M:Y38, CM:Y18

*All isolates fermented glucose, maltose, trehalose, sucrose, cellobiose, starch, and galactose. All isolates assimilated arabinose, rhamnose, sucrose, xylose, cellobiose, starch, and maltose*.

**Table 3:** Biolog identification of yeasts isolated from starters.

Yeast species	Probability (%)	Similarity	Distance	Status
*Pichia anomala*	0.943	0.683	4.185	Identified
*Pichia terricola*	0.974	0.768	3.182	Identified
*Pichia sydowiorum*	0.834	0.652	3.285	Identified
*Pichia onychis*	0.834	0.737	3.234	Identified
*Pichia guilliermondii*	0.834	0.652	3.223	Identified
*Pichia subpeliculum*	0.834	0.734	3.764	Identified
*Pichia trelalophila*	0.834	0.794	3.234	Identified
*Candia glabrata*	0.834	0.786	3.864	Identified
*Saccharomycopsis fibuligera*	0.934	0.739	3.123	Identified
*Zygosaccharomyces bailii*	0.834	0.783	3.652	Identified
*Phaffia rhodozyma*	0.734	0.768	3.223	Identified
*Debaryomyces*	0.934	0.752	3.682	Identified
*Debaryomyces castelli*	0.834	0.754	3.285	Identified
*Debaryomyces polymorphus*	0.834	0.783	2.876	Identified
*Issatchenkia orientalis*	0.834	0.656	3.987	Identified
*Saccharomyces cerevisiae*	0.834	0.765	3.243	Identified
*Rhodotorula bacarum*	0.834	0.784	2.239	Identified
*Rhodotorula aurantaea*	0.834	0.618	2.285	Identified
*Rhodotorula acheniorium*	0.916	0.742	3.947	Identified

**FIGURE 2:**
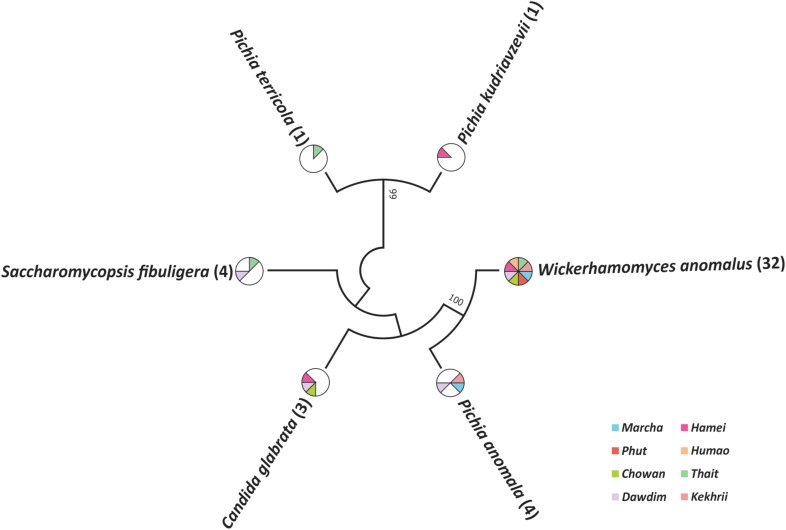
Molecular phylogenetic analysis of 46 yeast isolates recovered from a starters based on ITS region sequencing. The bootstrap consensus tree derived with 1000 replicates to neighbor-joining method and Kimura two-parameter model. Numbers on branches depict the percent occurrence of a given branch during 1000 replicates. The origin distribution patterns of these isolates were depicted in subsequent pi-charts.

Out of 386 isolates, 46 representative isolates were grouped based on colony appearance, cell shape, type of mycelia and ascospores, pellicle formation, nitrate reduction, and growth at 37 and 45°C. Precisely, species level identification was done with molecular methods by ITS-region gene sequence analysis. We found that all cultures were identified in six species only as: *Wickerhamomyces anomalus, Pichia anomala, Saccharomycopsis fibuligera, Pichia terricola, Pichia kudriavzevii*, and *Candida glabrata* which was reported in **Supplementary Data Sheet S2**. The average distributions in all starters and molecular phylogenetic relationship with neighbor-joining method were shown in **Figure 2**. From the sequencing results of ITS region gene; it was observed that species richness (R) was higher in *dawdim, hamei, thiat* than *marcha khekhrii, chowan*, and *phut* (**Table 4**). *Wickerhamomyces anomalus* was dominant in all starters. The Shannon index (H) of yeasts isolates was higher in *dawdim* than other starters (**Table 4**).

**Table 4:** Molecular characterization and identification results of 46 yeast strains from starters of North East India by PCR ITS1-5.8S ITS2.

Product	Isolate code	^a^AP	^b^H	^c^R	GenBank accession number	Species
*Marcha*	GM:29	554	0.642	2	KY605141	*Wickerhamomyces anomalus*
	GM:Y1	582	0.613		KY605153	*Wickerhamomyces anomalus*
	GM:Y5	548	0.623		KY605154	*Wickerhamomyces anomalus*
	GM:Y12	529	0.626		KY587129	*Pichia anomala*
	GM:Y29	483	0.625		KY587130	*Wickerhamomyces anomalus*
	GM:Y46	604	0.623		KY587131	*Wickerhamomyces anomalus*
	M:Y5	658	0.622		KY605150	*Wickerhamomyces anomalus*
*Thiat*	ST:Y21	793	6.000		KY605140	*Saccharomycopsis fibuligera*
	ST:Y6	705	0.911	3	KY605145	*Wickerhamomyces anomalus*
	ST:Y24	840	0.941		KY605146	*Pichia terricola*
	ST:Y15	624	0.921		KY605147	*Saccharomycopsis fibuligera*
	ST:Y12	702	0.901		KY605148	*Wickerhamomyces anomalus*
	ST:Y3	596	6.911		KY605149	*Wickerhamomyces anomalus*
	ST:Y49	661	0.921		KY626330	*Wickerhamomyces anomalus*
*Hamei*	M:Y8	661	0.911	3	KY587121	*Wickerhamomyces anomalus*
	HS:Y7	1031	0.921		KY626335	*Pichia kudriavzevii*
	AH:45	458	0.921		KY605155	*Candida glabrata*
	H:Y7	710	0.941		KY605152	*Pichia kudriavzevii*
*Huamo*	AS:Y3	515	0.441	1	KY587126	*Wickerhamomyces anomalus*
	AS:Y5	601	0.441		KY587127	*Wickerhamomyces anomalus*
	AS:Y7	594	0.401		KY587128	*Wickerhamomyces anomalus*
	AS:Y4	565	0.431		KY605162	*Wickerhamomyces anomalus*
*Chowan*	CH:Y28	801	0.621	2	KY605143	*Candida glabrata*
	CH:Y39	574	0.601		KY605144	*Wickerhamomyces anomalus*
	CX:44	258	0.621		KY605159	*Wickerhamomyces anomalus*
	CH:X26	594	0.611		KY605160	*Wickerhamomyces anomalus*
	CH:X39	918	0.631		KY626331	*Wickerhamomyces anomalus*
	CH:Y22	845	0.601		KY626334	*Wickerhamomyces anomalus*
*Phut*	ST:Y53	927	0.410	1	KY626332	*Wickerhamomyces anomalus*
	ST:Y20	919	0.400		KY626333	*Wickerhamomyces anomalus*
*Dawdim*	M:Y9	592	1.100	4	KY587136	*Wickerhamomyces anomalus*
	M:Y20	484	1.030		KY587137	*Wickerhamomyces anomalus*
	M:Y30	529	1.002		KY587138	*Candida glabrata*
	M:Y47	588	1.001		KY587139	*Wickerhamomyces anomalus*
	M:Y57	585	1.1 11		KY587140	*Wickerhamomyces anomalus*
	M:Y3	629	1.121		KY587119	*Wickerhamomyces anomalus*
	M:Y6	627	1.120		KY587120	*Pichia anomala*
	ST:Y15	692	1.120		KY605157	*Saccharomycopsis fibuligera*
	XT:Y20	610	1.131		KY605156	*Pichia anomala*
	XT:Y15	654	1.113		KY605147	*Saccharomycopsis fibuligera*
*Khekhrii*	K:Y8	558	0.630	2	KY605151	*Wickerhamomyces anomalus*
	K:Y20	589	0.600		KY605152	*Wickerhamomyces anomalus*
	K:Y18	529	0.601		KY587132	*Wickerhamomyces anomalus*
	K:Y27	599	0.611		KY587133	*Pichia anomala*
	K:Y38	604	0.620		KY587134	*Wickerhamomyces anomalus*
	K:Y45	599	0.612		KY587135	*Wickerhamomyces anomalus*

*^*a*^AP, arbitrary primers = sizes in base pairs; ^*b*^H, Shannon’s index; ^*c*^R, species richness. Only gene bank percent of strains with more than 90% were shown in the table*.

### Culture-Independent Approach

**FIGURE 3:**
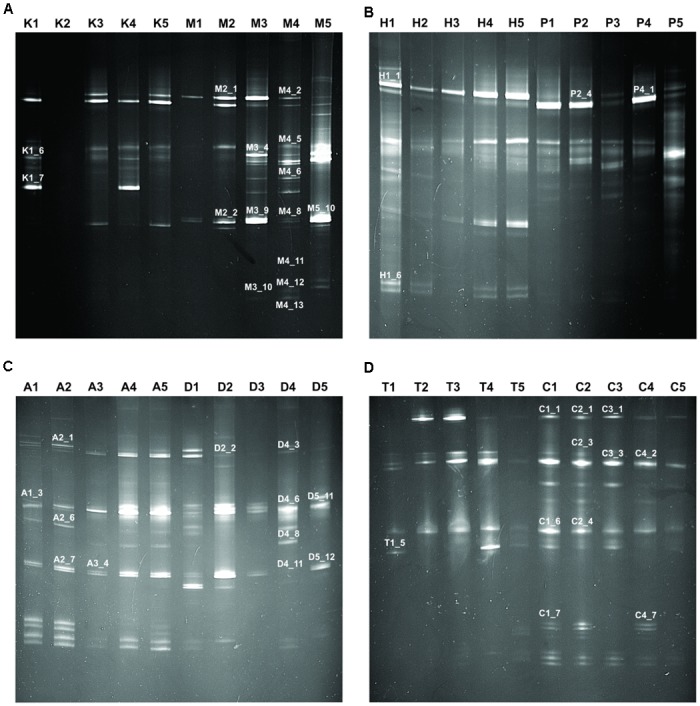
Fingerprint of PCR-DGGE analysis of different samples. Total 40 samples were taken for PCR-DGGE wherein five samples from each amylolytic starter used for fingerprinting. Samples and respective band patterns are demonstrated like: **(A)** *Khekrii* (K) and *Marcha* (M); **(B)** *Hamai* (H) and *Humao* (P); **(C)** *Phut* (A) and *Dawdim* (D); **(D)** *Thiat* (T) and *Chowan* (C). Representation of band numbers of respective bands on fingerprint were those which showed ≥98% sequence identity to GeneBank nucleotide database.

**FIGURE 4:**
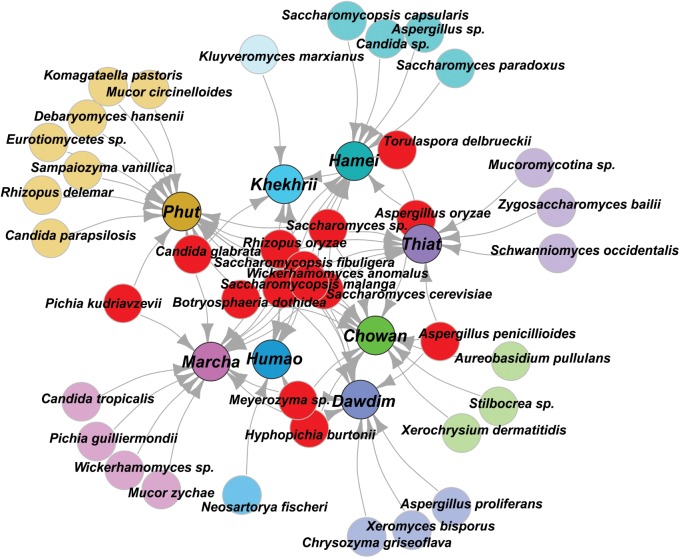
Graphical representation of all species identified in PCR-DGGE of 26SrRNA gene after sequencing. Shared species were represented in red color, and sample specific unique species were represented in respective colors to the starter samples and arrow indicated the origin distribution patterns of these isolates.

**FIGURE 5:**
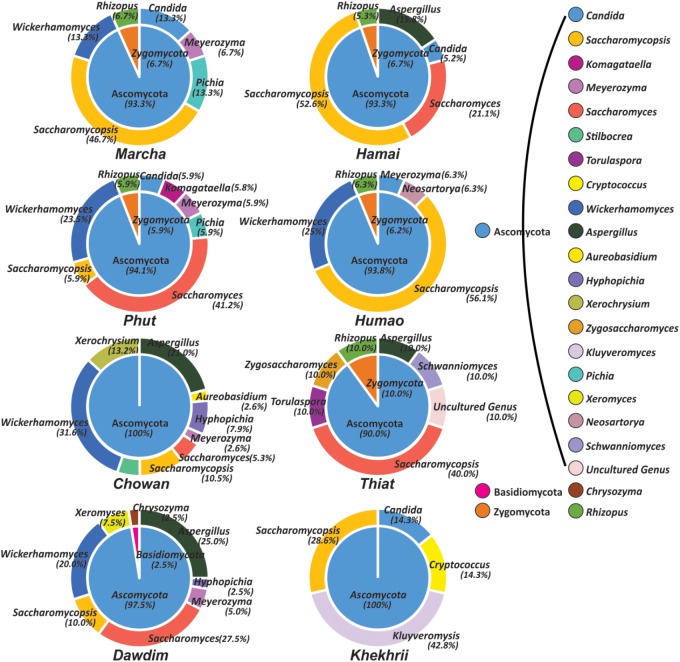
Genus and phylum level distribution of yeast and molds diversity in starters. Genus and respective phylum presented here was based on 98% identity cutoff value to the GeneBank database.

In this study, we targeted D1 and D2 domain of 26S rRNA gene (large ribosomal subunit) of fungi from 40 samples of starters using PCR-DGGE fingerprint analysis. We used NL-1 forward primer and a new LS2 reverse primer to amplify the portion of 26S rRNA gene. These primers amplified a product of approximately 250 bp covering most of the D1 expansion loop. In PCR-DGGE fingerprint, diversity map distributions in the form of band patterns of yeasts and molds had been observed in different starters (**Figure 3**). Total 202 DGGE bands were selected on the basis of visualizing the prominent and differential band patterns inside the gels, after analysis of raw sequenced data with the help of BLAST comparison in GenBank as presented in **Supplementary Data Sheet S2**. More than 98% similar identity with the closest species of yeasts and molds has different phylum and genus level distribution pattern in different starters (**Figure 4**). Interestingly, we observed the distinct species were more than the shared species and *phut* was found to have highest diversity (**Figure 5**).

**FIGURE 6:**
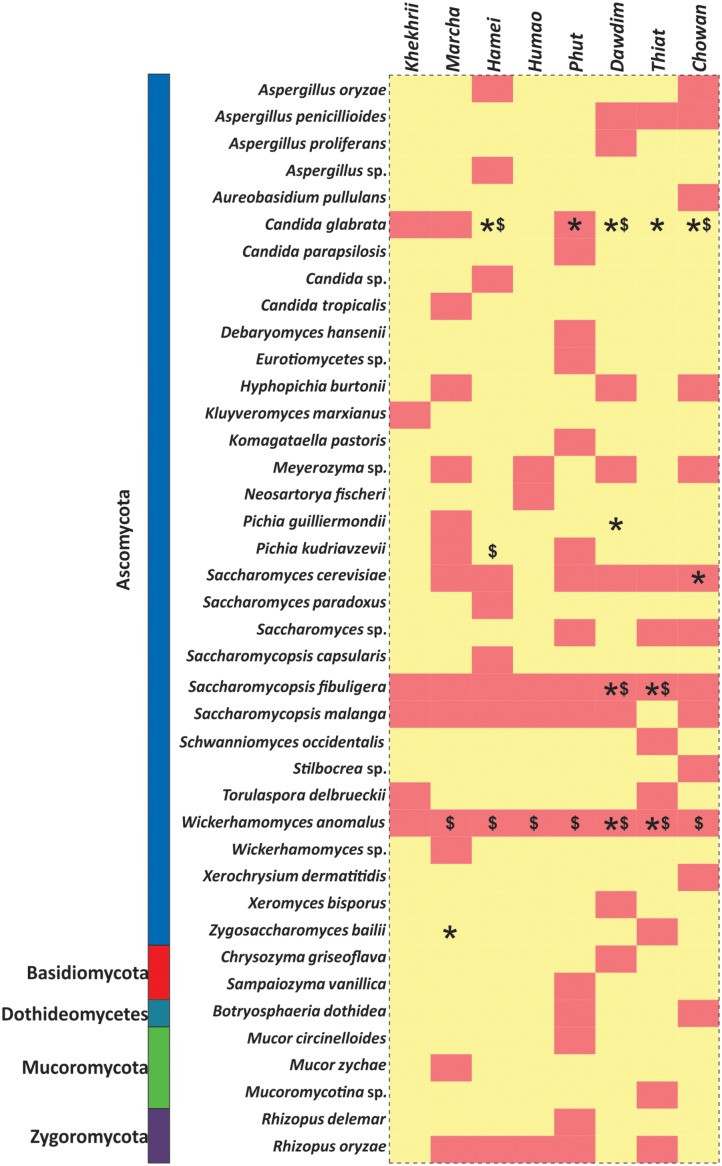
Heatmap showing the consensus species diversity observed during PCR-DGGE, Biolog identification hits and ITS region gene sequencing of yeast isolates. We used presence–absence value of PCR-DGGE species data to generate heatmap whereas red color indicates the presence and in other hand yellow color represents absence value. Other datasets were mapped over the heatmap like: Biolog identification (^∗^) and ITS-region gene sequencing of yeast isolates ($).

All these different techniques revealed the diversity and their differences of mycobiome species in different starters (**Figure 6**). Notably, the average distributions of yeasts in all samples were summarized as *Saccharomyces cerevisiae* (16.5%), *Saccharomycopsis fibuligera* (15.3%), *Wickerhamomyces anomalus* (11.3%), *S. malanga* (11.7%), *Kluyveromyces marxianus* (5.3%), *Meyerozyma* sp. (2.7%), *Candida glabrata* (2.7%), *Saccharomyces* sp. (1.3%), *Hyphopichia burtonii* (1.2%), *Schwanniomyces occidentalis* (1.1%), *Pichia kudriavzevi* (1.0%), *Torulaspora delbrueckii* (1.0%), *Zygosaccharomyces bailii* (1.0%), *Pichia guilliermondii* (1.0%), *Candida parapsilosis* (0.4%), *Komagataella pastoris* (0.3%), *S. capsularis* (0.6%), *S. Paradoxus* (0.6%), and *C. tropicalis* (0.1%). Similarly, the average distributions of molds in amylolytic starters were *Aspergillus penicillioides* (5.0%), *Rhizopus oryzae* (3.3%), sub-phylum: *Mucoromycotina* (2.1%), *Cryptococcus amylolentus* (1.7%), *Xerochrysium dermatitidis* (1.6%), *Aspergillus oryzae* (1.3%), *Neosartorya fischeri* (0.8%), *A. proliferans* (0.6%), *Chrysozyma griseoflava* (0.6%), *Stilbocrea*sp. *(*0.6%), *Mucor circinelloides* (0.5%), *Aureobasidium pullulans* (0.4%), and *Xeromyces bisporus* (0.3%).

## Discussion

Due to geographical locations, starters may have different and distinct mycobiome species diversity (Jeyaram et al., 2011). Going forward with this hypothesis, we examined and produced extensive surveillance report in different starters used in Indian alcoholic beverage production as an ethnic constituent. Results from Biolog system, where the profile of growth responses provides a metabolic fingerprint for each isolate (Praphailong et al., 1997), showed more diversity of yeasts in starters of North East India than phenotypic characterization based on probability and similarities index value. Even with high reliability rates, both phenotypic and Biolog tests did not coincide with the molecular reference tests for the majority of the isolates: when the identification results by Biolog were compared to 18S rRNA gene sequencing and species-specific PCR reactions (Nisiotou and Nychas, 2007). It has been previously reported that the ITS region gene analysis is a reliable routine technique for the differentiation of yeasts at species level (Clemente-Jimenez et al., 2004; Combina et al., 2005; Zott et al., 2008). Considering that species-specific PCR protocols target specific genes of genera and species, the reliability of ITS region gene sequences was considered to be 100% (Moraes et al., 2013). Another advantage of molecular culture-dependent method, which includes ITS, is that it allows a collection of pure cultures that may be used for further selection of suitable yeast strains to improve quality of alcoholic beverages (Lv et al., 2013).

In this study, *Wickerhamomyces anomalus, Pichia anomala, Saccharomycopsis fibuligera*, and *Candida glabrata* were identified in starters using ITS analysis. The previous studies also reported *Candida glabrata, Pichia anomala*, and *Saccharomycopsis fibuligera* from *marcha* based on 18S rDNA sequences (Tsuyoshi et al., 2005). It has been reported that *Candida glabrata*, which is a moderate alcohol producer, has also been recovered in *kodo ko jaanr*, ethnic fermented finger millet beverage prepared by using *marcha* (Thapa and Tamang, 2004) and some traditional Vietnamese starters (Dung et al., 2007), indicating that it is involved in alcohol production. Non*-Saccharomyces* yeasts may contribute to flavor or aroma formation in the alcoholic beverage (Rojas et al., 2001; Fleet, 2003; Moreira et al., 2005; Dung et al., 2006; Jolly et al., 2017). *Saccharomycopsis fibuligera, Saccharomyces cerevisiae, Wickerhamomyces anomala, Pichia* sp., and *Candida* sp. are the most common yeasts present in rice-based starters of Asia (Lee and Fujio, 1999; Xie et al., 2007; Jeyaram et al., 2008). Interestingly, *Wickerhamomyces anomalus*, probably the most abundant yeast, was reported for the first time from all the eight amylolytic starters of North East India using ITS-PCR method. The multiple sequence alignment of the ITS region gene sequences of *Wickerhamomyces anomalus* may be used for many purposes including inferring the presence of ancestral relationships between the sequences (Rampersad, 2014). It may be noted that protein sequences that are structurally very similar can be evolutionarily distant which is referred to as distant homology (Li and Durbin, 2010).

Genomic DNA extracted directly from samples of dried starters of India using the PCR-DGGE tools showed diversity of yeasts *Wickerhamomyces anomalus, Saccharomyces cerevisiae, S. malanga, S. paradoxus, Saccharomycopsis fibuligera, Sm. Capsularis, Candida glabrata, C. tropicalis, Meyerozyma* sp., *Pichia guilliermondii*, and *P. kudriavzevi*. Some researchers have reported the microbial community in some traditionally prepared dried starters for production of alcoholic beverages using PCR-DGGE analysis such as principal amylase-producer yeast *Sm. fibuligera* and ethanol-producers *S. cerevisiae* in *banh men* of Vietnam (Thanh et al., 2008), *nuruk* of Korea (Jung et al., 2012), and *yaa qu* and *hong qu* of China (Lv et al., 2012, 2013; Chen et al., 2014), respectively. *Sm. fibuligera* secretes considerable amount of α-amylase, glucoamylase, acid proteases, and β-glucosidase, which are applied in the fermentation industry (Chi et al., 2009).

The dominance of *S. cerevisiae* in *marcha, thiat, dawdim*, and *phut* might be due to its competitive growth in the presence of fermentable sugars and its ethanol tolerance may be due to fast growth during various alcoholic fermentations (Dung et al., 2006, 2007; Jeyaram et al., 2008). *S. cerevisiae* has also found to be one of the dominant yeasts in all starters of North East India, because of its competitive growth under strict anaerobic conditions and its tolerance to ethanol (Romano et al., 2006). *Wickerhamomyces anomalus*, a regular component in several types of Asia-Pacific alcohol fermentation starters (Limtong et al., 2002; Thanh et al., 2008), was detected in all analyzed samples. *P. guilliermondii* which was observed in *marcha* was also reported from wheat-based *qu* for Chinese Shaoxing rice wine (Xie et al., 2007) and *hamei* of Manipur in India (Jeyaram et al., 2008), which can produce volatile phenols and esters in the initial stages of alcoholic fermentation (Moreira et al., 2005). *Pichia kudriavzevii, Wickerhamomyces anomalus, S. malanga, Kluyveromyces marxianus, Torulaspora delbrueckii, Hyphopichia burtonii, S. capsularis*, and *Debaryomyces hansenii* were also reported from other Asian starters for the production of flavor and ethanol (Dung et al., 2006; Xie et al., 2007; Thanh et al., 2008; Zhang et al., 2008; Jung et al., 2012; Lv et al., 2013; Chen et al., 2014). *Zygosaccharomyces bailii* is widely present in various food fermentations, such as wine, tea, and vinegar fermentations (Garavaglia et al., 2015), and also produced various flavor compounds including alcohol in Chinese *Maotai* liquor (Xu et al., 2017).

In *chowan*, few pathogenic fungi were also detected such as *Xerochrysium dermatitidis*, which is a pathogenic fungus causing skin diseases (Pitt et al., 2013); and *Aureobasidium pullulans*, a ubiquitous black, yeast-like human fungal pathogen found in soil, water, air, and limestone (Chan et al., 2011). These pathogenic fungi may be contaminated through various raw substrates including wild herbs, water, etc. during crude preparation of *chowan* by village people in Tripura. The presence of sub-phylum: *Mucoromycotina*, which is the earliest mutualistic symbiosis fungus with *Haplomitriopsida* liverworts (Field et al., 2015),probably passed through the plants used during preparation of *thiat.*

Besides yeast community, some molds *Rhizopus* spp. and *Aspergillus* spp. were also detected by PCR-DGGE analysis in starters except in *khekhrii* samples of Nagaland (prepared by naturally fermenting germinated sprouted rice grains). Species of *Rhizopus* spp. and *Aspergillus* were reported from many Asian amylolytic starters (Tamang et al., 1988; Oda et al., 2006; Yang et al., 2013; Zhu and Tramper, 2013). The distributions of yeasts communities in amylolytic starters of North East India were higher in comparison to molds, this may be due to low temperatures of that particular environment in North East India and also the substrates used for fermentation (Chi et al., 2009). These traditional starters are the result of long-term selection for preserving and cultivation the amylolytic and alcohol-producing native yeasts and fungi by ethnic people which has been practicing the traditional process for centuries (Tamang, 2010; Londoño-Hernández et al., 2017). The DGGE analysis has some disadvantages due to its inability to determine the relative abundance of dominant species, differentiate between viable and nonviable cells, and difficulties in interpretation of multi-bands (Nam et al., 2012; Dolci et al., 2015). Besides, DNA extraction efficiencies vary between microorganisms since DGGE band intensity is not always correlated with population density (Ercolini, 2004; Prakitchaiwattana et al., 2004; Lv et al., 2013). Sub-culturing or back sloping of desirable inocula from previous batch during the traditional preparation of starters under uncontrolled fermentation may pose health risks (Rossetti et al., 2009). However, combination of culture-dependent and -independent analysis may be used to assess the safety of the microbiota associated with spontaneous/natural fermentation that may help to predict the possible risks for human health (Capozzi et al., 2007; Van Hijum et al., 2013).

## Conclusion

Starter making technology reflects the traditional method of “sub-culturing” of desirable inocula from previous batch to new culture using rice as base substrates by back-sloping, in North East India. Selection of ethnic starters from different geographical regions with diverse mycobiome is gaining the importance of species diversity as indigenous property. We performed one of the successful trials to find out the mycobiome associated with eight different dried starters of North East India analyzed by ITS-PCR and PCR-DGGE techniques. These results may enrich our knowledge of cultivable indigenous mycobiota present in the starters (amylolytic and alcoholic) of Asia that may be used to promote the production technology of unique ethnic alcoholic beverages high quality and typical attributes; moreover, data of starters of India can be used as reference data base for the further research.

## Author Contributions

SS contributed to this present work as a part of his research work. MS and KJ helped and assisted in some molecular work Bioinformatics analysis. AS, YS, and JT framed and prepared this paper critically with final approval of JT.

## Conflict of Interest Statement

The authors declare that the research was conducted in the absence of any commercial or financial relationships that could be construed as a potential conflict of interest.

## Footnotes

1 http://www.northeasttourism.gov.in

2 http://www.technelysium.com.au/chromas.html

3 http://blast.ncbi.nlm.nih.gov/Blast.cgi

## Supplementary Material

DATA SHEET S1 GenBank accessions number of identified species of yeasts.

DATA SHEET S2 Biolog identification of yeast strains isolated from different amylolytic starters of North East India.
