# Supplementary material for: Diversity of Yeasts and Molds by Culture-Dependent and Culture-Independent Methods for Mycobiome Surveillance of Traditionally Prepared Dried Starters for the Production of Indian Alcoholic Beverages
Source: Front Microbiol. 2018 Sep 26;9:2237. doi: 10.3389/fmicb.2018.02237 (PMC6169615; doi:10.3389/fmicb.2018.02237)
Supplement: DATA SHEET S1 — GenBank accessions number of identified species of yeasts. [file Data_Sheet_1.DOCX]

| Sl. No | isolate code | Identified strains | Samples |
| --- | --- | --- | --- |
| 1 | CHY28 | Debromyces castelii | chowan |
| 2 | CHY39 | Pichia jydowiorum | chowan |
| 3 | CX44 | Phichia onychis | chowan |
| 4 | CHX26 | Debromyces polymorphus | chowan |
| 5 | CHX39 | Issatchenkia orientalis | chowan |
| 6 | CHY22 | Saccharomyces ceriviciae | chowan |
| 7 | CHY38 | Pichia anomalus | chowan |
| 8 | CHY28 | Candida glabrata | chowan |
| 9 | CHY39 | Pichia anomalus | chowan |
| 10 | MY15 | Pichia anomalus | dawdim |
| 11 | MY9 | Rhodotorula aurantaea | dawdim |
| 12 | MY20 | Pichia anomalus | dawdim |
| 13 | MY30 | Pichia onychis | dawdim |
| 14 | MY47 | Pichia anomalus | dawdim |
| 15 | MY57 | Candia glabrata | dawdim |
| 16 | MY3 | Pichia anomalus | dawdim |
| 17 | MY6 | Pichia guillermondii | dawdim |
| 18 | STY15 | Pichia anomalus | dawdim |
| 19 | MY5 | Pichia anomalus | dawdim |
| 20 | MY3 | Pichia anomalus | dawidim |
| 21 | MY6 | Pichhia anomalus | dawidim |
| 22 | MY8 | Pichia anomalus | dawidim |
| 23 | MY8 | Saccharomycopsis fibuligera | dwadim |
| 24 | HSY7 | Pichia anomalus | hamei |
| 25 | AH45 | Pichia anomalus | hamei |
| 26 | HSY7 | Candida glabrata | hamei |
| 27 | ASY3 | Pichia anomalus | humao |
| 28 | ASY5 | Pichia anomalus | humao |
| 29 | ASY5 | Pichia terricola | humao |
| 30 | ASY4 | Pichia anomalus | humao |
| 31 | KY8 | Pichia anomalus | khekhriii |
| 32 | KY20 | Rhhodotorula acheniorium | khekhriii |
| 33 | KY18 | Pichia anomalus | khekhriii |
| 34 | KY27 | Pichia subpeliculum | khekhriii |
| 35 | KY38 | Pichia trelalophila | khekhriii |
| 36 | KY45 | Endomyces fibuligera | khekhriii |
| 37 | GMY1 | Zygosaccharomyces bailii | marcha |
| 38 | GMY5 | Pichia anomalus | marcha |
| 39 | GMY12 | Rhodotolura bacarum | marcha |
| 40 | GMY29 | Endomyces fibuligera | marcha |
| 41 | GMY46 | Phaphia rhodozymas | marcha |
| 42 | GM29 | Pichia anomalus | marcha |
| 43 | GM29 | Pichia anomalus | marcha |
| 44 | XTY20 | Pichia sydowiorum | phut |
| 45 | STY15 | Candida glabrata | phut |
| 46 | STY20 | Pichia anomalus | phut |
| 47 | STY21 | Pichia anomalus | phut |
| 49 | STY21 | Candida glabrata | thiat |
| 49 | STY6 | Candida glabrata | thiat |
| 50 | STY24 | Pichia anomalus | thiat |
| 51 | STY15 | Pichia anomalus | thiat |
| 52 | STY12 | Debromyces | thiat |
| 53 | STY3 | Debromyces castelii | thiat |
| 54 | STY49 | Debromyces polymorphus | thiat |
| 55 | STY49 | Candida glabrata | thiat |
| 56 | STY6 | Pichia anomalus | thiat |
| 57 | STY24 | Pichioa terricola | thiat |
| 59 | STY15 | Saccharomycopsis fibuligera | thiat |
| 59 | STY12 | Pichia anomalus | thiat |
| 60 | STY3 | Pichia anomalus | thiat |
