# Supplementary material for: Diversity of Yeasts and Molds by Culture-Dependent and Culture-Independent Methods for Mycobiome Surveillance of Traditionally Prepared Dried Starters for the Production of Indian Alcoholic Beverages
Source: Front Microbiol. 2018 Sep 26;9:2237. doi: 10.3389/fmicb.2018.02237 (PMC6169615; doi:10.3389/fmicb.2018.02237)
Supplement: DATA SHEET S2 — Biolog identification of yeast strains isolated from different amylolytic starters of North East India. [file Data_Sheet_2.DOCX]

| Sl. No | Isolate code | GenBank accessions | Identified Species | Samples |
| --- | --- | --- | --- | --- |
| 1 | GM29 | KY605141 | Wickerhamomyces anomalus | Marcha |
| 2 | GMY1 | KY605153 | Wickerhamomyces anomalus | Marcha |
| 3 | GMY5 | KY605154 | Wickerhamomyces anomalus | Marcha |
| 4 | GMY12 | KY587129 | Pichia anomala | Marcha |
| 5 | GMY29 | KY587130 | Wickerhamomyces anomalus | Marcha |
| 6 | GMY46 | KY587131 | Wickerhamomyces anomalus | Marcha |
| 7 | MY5 | KY605150 | Wickerhamomyces anomalus | Marcha |
| 8 | STY21 | KY605140 | Saccharomycopsis fibuligera | Thiat |
| 9 | STY6 | KY605145 | Wickerhamomyces anomalus | Thiat |
| 10 | STY24 | KY605146 | Pichia terricola | Thiat |
| 11 | STY15 | KY605147 | Saccharomycopsis fibuligera | Thiat |
| 12 | STY12 | KY605148 | Wickerhamomyces anomalus | Thiat |
| 13 | STY3 | KY605149 | Wickerhamomyces anomalus | Thiat |
| 14 | STY49 | KY626330 | Wickerhamomyces anomalus | Thiat |
| 15 | MY8 | KY587121 | Wickerhamomyces anomalus | hamei |
| 16 | HSY7 | KY626335 | Pichia kudriavzevii | hamei |
| 17 | AH45 | KY605155 | Candida glabrata | hamei |
| 18 | HY7 | KY605142 | Pichia kudriavzevii | hamei |
| 19 | ASY3 | KY587126 | Wickerhamomyces anomalus | humao |
| 20 | ASY5 | KY587127 | Wickerhamomyces anomalus | humao |
| 21 | ASY7 | KY587128 | Wickerhamomyces anomalus | huamo |
| 22 | ASY4 | KY605162 | Wickerhamomyces anomalus | huamo |
| 23 | CHY28 | KY605143 | Candida glabrata | chowan |
| 24 | CHY39 | KY605144 | Wickerhamomyces anomalus | chowan |
| 25 | CX44 | KY605159 | Wickerhamomyces anomalus | chowan |
| 26 | CHX26 | KY605160 | Wickerhamomyces anomalus | chowan |
| 27 | CHX39 | KY626331 | Wickerhamomyces anomalus | chowan |
| 28 | CHY22 | KY626334 | Wickerhamomyces anomalus | chowan |
| 29 | STY53 | KY626332 | Wickerhamomyces anomalus | phut |
| 30 | STY20 | KY626333 | Wickerhamomyces anomalus | phut |
| 31 | MY9 | KY587136 | Wickerhamomyces anomalus | dawdim |
| 32 | MY20 | KY587137 | Wickerhamomyces anomalus | dawdim |
| 33 | MY30 | KY587138 | Candida glabrata | dawdim |
| 34 | MY47 | KY587139 | Wickerhamomyces anomalus | dawdim |
| 35 | MY57 | KY587140 | Wickerhamomyces anomalus | dawdim |
| 36 | MY3 | KY587119 | Wickerhamomyces anomalus | dawdim |
| 37 | MY6 | KY587120 | Pichia anomala | dawdim |
| 38 | STY15 | KY605157 | Saccharomycopsis fibuligera | dawdim |
| 39 | XTY20 | KY605156 | Pichia anomala | dawdim |
| 40 | XTY15 | KY605147 | Saccharomycopsis fibuligera | dawdim |
| 41 | KY8 | KY605151 | Wickerhamomyces anomalus | khekhrii |
| 42 | KY20 | KY605152 | Wickerhamomyces anomalus | khekhrii |
| 43 | KY18 | KY587132 | Wickerhamomyces anomalus | khekhrii |
| 44 | KY27 | KY587133 | Pichia anomala | khekhrii |
| 45 | KY38 | KY587134 | Wickerhamomyces anomalus | khekhrii |
| 46 | KY45 | KY587135 | Wickerhamomyces anomalus | khekhrii |
